# Differential Metabolisms of Green Leaf Volatiles in Injured and Intact Parts of a Wounded Leaf Meet Distinct Ecophysiological Requirements

**DOI:** 10.1371/journal.pone.0036433

**Published:** 2012-04-30

**Authors:** Kenji Matsui, Kohichi Sugimoto, Jun'ichi Mano, Rika Ozawa, Junji Takabayashi

**Affiliations:** 1 Department of Biological Chemistry, Faculty of Agriculture, Yamaguchi University, Yamaguchi, Japan; 2 Center for Ecological Research, Kyoto University, Ohtsu, Japan; 3 Science Research Center, Yamaguchi University, Yamaguchi, Japan; 4 Department of Applied Molecular Bioscience, Graduate School of Medicine, Yamaguchi University, Yamaguchi, Japan; Max Planck Institute for Chemical Ecology, Germany

## Abstract

Almost all terrestrial plants produce green leaf volatiles (GLVs), consisting of six-carbon (C6) aldehydes, alcohols and their esters, after mechanical wounding. C6 aldehydes deter enemies, but C6 alcohols and esters are rather inert. In this study, we address why the ability to produce various GLVs in wounded plant tissues has been conserved in the plant kingdom. The major product in completely disrupted *Arabidopsis* leaf tissues was (*Z*)-3-hexenal, while (*Z*)-3-hexenol and (*Z*)-3-hexenyl acetate were the main products formed in the intact parts of partially wounded leaves. ^13^C-labeled C6 aldehydes placed on the disrupted part of a wounded leaf diffused into neighboring intact tissues and were reduced to C6 alcohols. The reduction of the aldehydes to alcohols was catalyzed by an NADPH-dependent reductase. When NADPH was supplemented to disrupted tissues, C6 aldehydes were reduced to C6 alcohols, indicating that C6 aldehydes accumulated because of insufficient NADPH. When the leaves were exposed to higher doses of C6 aldehydes, however, a substantial fraction of C6 aldehydes persisted in the leaves and damaged them, indicating potential toxicity of C6 aldehydes to the leaf cells. Thus, the production of C6 aldehydes and their differential metabolisms in wounded leaves has dual benefits. In disrupted tissues, C6 aldehydes and their *α*,*β*-unsaturated aldehyde derivatives accumulate to deter invaders. In intact cells, the aldehydes are reduced to minimize self-toxicity and allow healthy cells to survive. The metabolism of GLVs is thus efficiently designed to meet ecophysiological requirements of the microenvironments within a wounded leaf.

## Introduction

**Figure 1:**
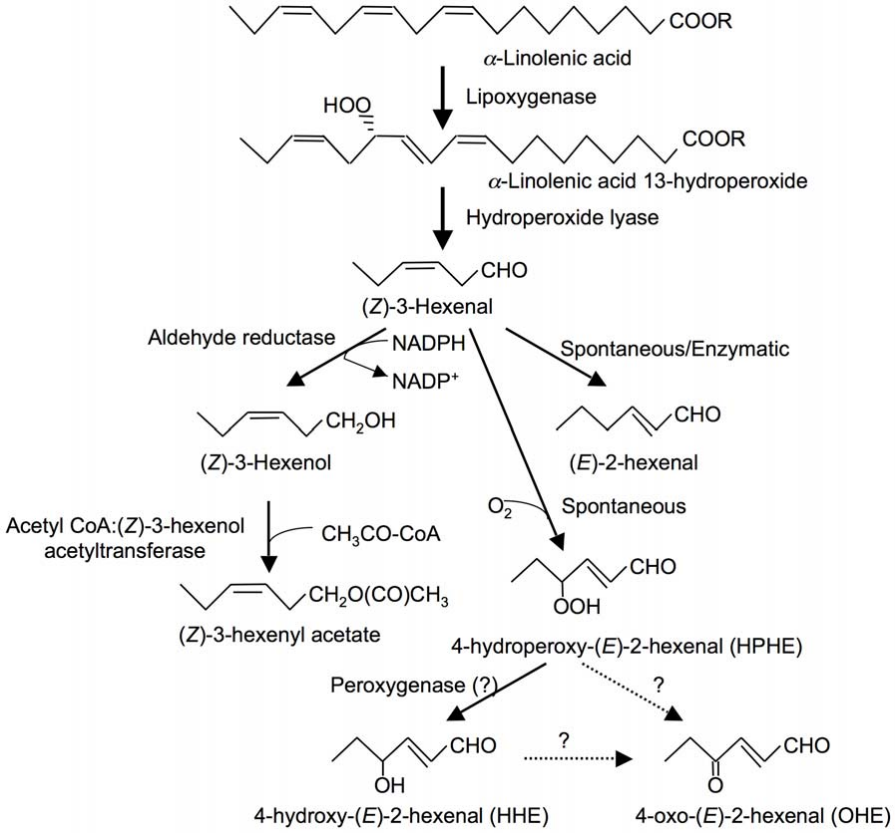
Biochemical pathway for the formation of (*Z*)-3-hexenal and its related compounds. In the biochemical pathway for formation of (*Z*)-3-hexenal and its related compounds, lipoxygenase adds dioxygen at position 13 of linolenic acid to produce linolenic acid 13-hydroperoxide. The hydroperoxide is cleaved by hydroperoxide lyase at the C12–C13 bond to produce (*Z*)-3-hexenal, which can be reduced to form (*Z*)-3-hexenol. A portion of (*Z*)-3-hexenol is further converted to (*Z*)-3-hexenyl acetate. In some plants, (*Z*)-3-hexenal is converted to (*E*)-2-hexenal spontaneously or enzymatically. (Z)-3-Hexenal is spontaneously oxygenated to form 4-hydroperoxy-(*E*)-2-hexenal, 4-hydroxy-(*E*)-2-hexenal or 4-oxo-(*E*)-2-hexenal.

Green leaf volatiles (GLVs), which consist of six-carbon (C6) aldehydes, alcohols, and their esters, are ubiquitous in the leaves of most plants [1]. The biosynthetic pathways that produce GLVs (Fig. 1) are widespread in the plant kingdom. Lipoxygenase adds dioxygen at position 13 of linolenic acid to produce linolenic acid 13-hydroperoxide. The hydroperoxide is cleaved by hydroperoxide lyase (HPL) at the C12–C13 bond to produce two carbonyl compounds. One of the primary products of HPL, (*Z*)-3-hexenal, can be reduced to form (*Z*)-3-hexenol. A portion of (*Z*)-3-hexenol is further converted to (*Z*)-3-hexenyl acetate by acetyl-CoA∶(*Z*)-3-hexenol acetyltransferase [2]. In some plants, (*Z*)-3-hexenal is converted to (*E*)-2-hexenal spontaneously or enzymatically [1], [3], [4]. A portion of (*E*)-2-hexenal, which has electrophilic properties because of its *α*,*β*-unsaturated carbonyl moiety, reacts with glutathione to form a glutathione-conjugate [5]. When linoleic acid is the starting material, this enzyme system catalyzes the formation of *n*-hexanal, *n*-hexanol, and *n*-hexyl acetate. Biosynthetic reactions that rely on reducing equivalents and acetyl-CoA are rather costly; thus, when plants convert (*Z*)-3-hexenal to (*Z*)-3-hexenol and (*Z*)-3-hexenyl acetate, they do so at the expense of materials that would otherwise be useful for growth.

GLVs are formed rapidly after the disruption of plant tissues [2], [4]. The physiological significance of the rapid formation of (*Z*)-3-hexenal has been discussed in the context of defense against biotic stresses [6], [7], [8], [9]. In fact, insecticidal, fungicidal, and bactericidal activities have been reported for (*Z*)-3-hexenal and its related aldehydes [6], [7], [8], [9]. Aphids (*Myzus persicae* (Sulzer)) feeding on transgenic potato plants with suppressed HPL activity displayed higher fecundity, suggesting that the C6 aldehydes formed by HPL were involved in a direct defense response against these insects [10]. Overexpression of HPL in *Arabidopsis* enhanced its direct defense response against the necrotrophic fungal pathogen *Botrytis cinerea* (De Bary) Whetzel, whereas antisense suppression of HPL resulted in higher susceptibility to the pathogen [11]. The susceptibility of HPL-deficient *Arabidopsis* to *B. cinerea* was largely attributed to the lower accumulations of (*Z*)-3-hexenal and (*E*)-2-hexenal after infection [8]. Necrotrophic fungal pathogens grow out from the infected tissues as they disrupt the plant cells [12], which is inevitably associated with the accumulation of (*Z*)-3-hexenal in the tissues. These results suggest that the rapid formation of (*Z*)-3-hexenal is involved in a direct defense against some herbivores and pathogens.

Furthermore, GLVs function as airborne infochemicals in specific plant–herbivore, plant–carnivore, and plant–plant relationships. Some herbivorous and carnivorous arthropods use GLVs as infochemicals in their foraging behavior. For example, the herbivorous cockchafers (*Melolontha melolontha* L.) are attracted to (*Z*)-3-hexenol [13]. (*Z*)-3-Hexenol is also a cue for the parasitic wasp, *Opius dissitus* Muesebeck, to search for its host herbivore, *Liriomyza huidobrensis* (Blanchard) [14]. (*Z*)-3-Hexenol, (*Z*)-3-hexenyl acetate, and (*E*)-2-hexenal, all of which are present in odor blends either from *Tetranychus urticae* C. L. Koch–infested or physically-damaged bean leaves, attract the predator *Neoseiulus californicus* (McGregor) [15]. *Cotesia glomerata* (L.), a parasitic wasp of cabbage white butterfly larvae, is attracted by (*Z*)-3-hexenyl acetate and (*E*)-2-hexenal emitted by infested plants [16]. A factor in the oral secretions of *Manduca sexta* (L.) larvae isomerizes (*Z*)-3-hexenal to (*E*)-2-hexenal, and as a result, lowers the *Z*/*E* ratio of the GLVs, leading to greater foraging efficiency of predators in nature [4]. Furthermore, GLVs such as (*Z*)-3-hexenol and/or (*Z*)-3-hexenyl acetate are involved in plant–plant signaling [17]–[21]. The diverse ecological functions of GLVs are the result of evolutionary interactions between plants and their pathogens, herbivores and their carnivores, and neighboring plants. Thus, GLVs convey context-dependent multifunctional information in nature.

However, the fundamental question of why plants produce not only (*Z*)-3-hexenal, but also the more costly (*Z*)-3-hexenol and (*Z*)-3-hexenyl acetate remains unanswered. The ability to form diverse GLVs has been conserved in the evolution and diversification of higher plants. This extreme conservation cannot be explained by context-dependent ecological interactions between plants and other organisms. Rather, the ubiquity of GLVs might be addressed in light of their direct defensive functions, i.e., their toxicity, and of their fates, i.e., the biosynthetic pathway. Here, we address the reason(s) why plants produce diverse blends of GLVs by considering the fates of GLVs in leaf tissue and their potential auto-toxicity.

## Results

### GLVs formed after disruption

**Figure 2:**
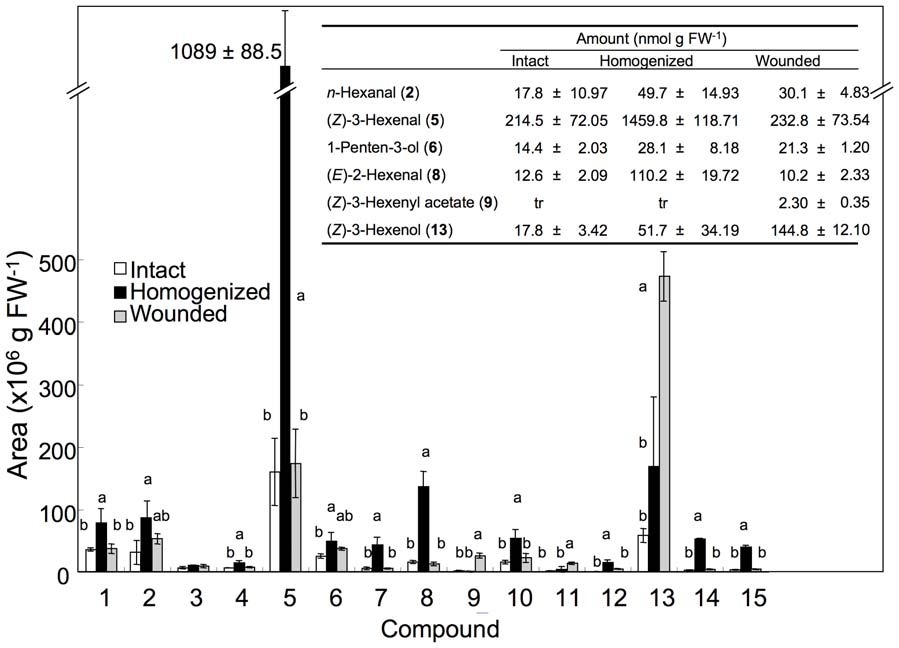
Amounts of volatile compounds formed by intact, disrupted, and partially wounded leaves of *Arabidopsis* (*No-0*). Peaks: 1, 1-penten-3-one; 2, *n*-hexanal; 3, 2-pentenal*; 4, 2-pentenal* (probably a geometrical isomer of compound 3); 5, (*Z*)-3-hexenal; 6, 1-penten-3-ol; 7, (*Z*)-2-hexenal*; 8, (*E*)-2-hexenal; 9, (*Z*)-3-hexenyl acetate; 10, (*Z*)-2-pentenol; 11, *n*-hexanol; 12, (*E*)-3-hexenol*; 13, (*Z*)-3-hexenol; 14, 4-oxo-(*E*)-2-hexenal*; 15, 4-hydroxy-(*E*)-2-hexenal. Asterisks indicate compounds that were tentatively identified based on MS data (according to the NIST library). Compounds without asterisks were identified from retention indices and MS of corresponding authentic specimens. Inset: amounts of major volatile compounds quantified from corresponding calibration curves. Error bars represent SE (*n* = 3). Data were analyzed by Tukey's test. Different letters above the bars indicate significant differences among treatments for each compound (*P*<0.05).

In intact *Arabidopsis* (*No-0*) leaves, we detected low amounts of GLVs and C5 compounds, such as 1-penten-3-one, 1-penten-3-ol, and (*Z*)-2-pentenol (Fig. 2, Fig. S1). When the leaves were completely disrupted and incubated for 5 min, there was extensive formation of GLVs. The most prominent increase was observed for (*Z*)-3-hexenal, which reached a concentration of approximately 1.5 µmol g fresh weight (FW)^−1^. The total amounts of free and esterified C18∶3 and C16∶3 in the intact *Arabidopsis* leaves were ca. 5.5 µmol g FW^−1^. Therefore, approximately 27.3% of the trienoic acids in the leaf tissues were converted into (*Z*)-3-hexenal within 5 min of complete disruption. (*E*)-2-Hexenal, which was thought to be formed via isomerization of (*Z*)-3-hexenal, also increased, and accounted for 6.8% of all C6 compounds formed from trienoic fatty acids. The amount of (*Z*)-3-hexenol doubled, but only a trace amount of (*Z*)-3-hexenyl acetate was detected after complete disruption of the leaf tissue. 4-Hydroxy-(*E*)-2-hexenal (HHE) and 4-oxo-(*E*)-2-hexenal (OHE) were detected for the first time in *Arabidopsis* (Fig. S2). OHE was mostly derived from pyrolysis of 4-hydroperoxy-(*E*)-2-hexenal (HPHE) during GC-MS analyses, because the addition of triphenylphosphine dissipated OHE and enlarged the peak of HHE. This is hereafter denoted OHE/HPHE.

When approximately 20% of the leaf area was mechanically wounded by crushing with forceps, (*Z*)-3-hexenol was the main volatile product formed (Fig. 2), whereas the amount of (*Z*)-3-hexenal was similar to that found in intact leaves. We assumed that the (*Z*)-3-hexenal produced in the disrupted tissues was largely converted into (*Z*)-3-hexenol. A significant amount of (*Z*)-3-hexenyl acetate was also detected. The amounts of other aldehydes such as (*E*)-2-hexenal, OHE/HPHE, and HHE were low and were nearly the same as those found in intact leaves. These results indicated that isomerization and oxidation of (*Z*)-3-hexenal did not occur to any significant extent, even though (*Z*)-3-hexenal should have been formed in the disrupted tissues as a primary product of the GLV pathway, as was observed in completely disrupted leaf tissues. The lack of an increase in (*Z*)-3-hexenal and these (*Z*)-3-hexenal derivatives can be attributed to the rapid conversion of (*Z*)-3-hexenal to (*Z*)-3-hexenol. Taken together, these results suggested that remaining intact cells in the injured leaf metabolized (*Z*)-3-hexenal in a different way.

The total amount of GLVs (420 nmol g FW^−1^) detected from partially wounded leaves was ca. 25% of that measured from homogenized leaves (1,671 nmol g FW^−1^). Because ca. 20% of the total leaf area was wounded, we assumed that GLVs detected in the wounded leaves were mostly derived from aldehydes formed in the disrupted part of the wounded leaves, and that the intact part of wounded leaves contributed little to the formation of GLVs. After mechanically disrupting half of a *No-0* leaf, the wounded part was carefully excised from the plant after 5 min. The total amount of GLVs found in the remaining part was as little as 55 nmol g FW^−1^. This observation supported the assumption that the aldehyde forms of GLVs were produced predominantly by the disrupted tissues of the partially wounded leaves, but not (or very little) by the neighboring, intact tissues.

### Diffusion of (*Z*)-3-hexenal

Based on the differences in the compositions of GLVs produced by completely disrupted and partly wounded (i.e., with a patchy distribution of intact and completely disrupted tissues) *Arabidopsis* leaves, we assumed that the (*Z*)-3-hexenal formed in the disrupted tissues diffused into neighboring intact tissues, where the aldehyde was immediately reduced to (*Z*)-3-hexenol. Some of the (*Z*)-3-hexenol formed in this reaction could then be further acetylated to (*Z*)-3-hexenyl acetate. This scenario would be possible if there were high activity for reduction of (*Z*)-3-hexenal to (*Z*)-3-hexenol in intact tissues, but not in disrupted tissues.

**Figure 3:**
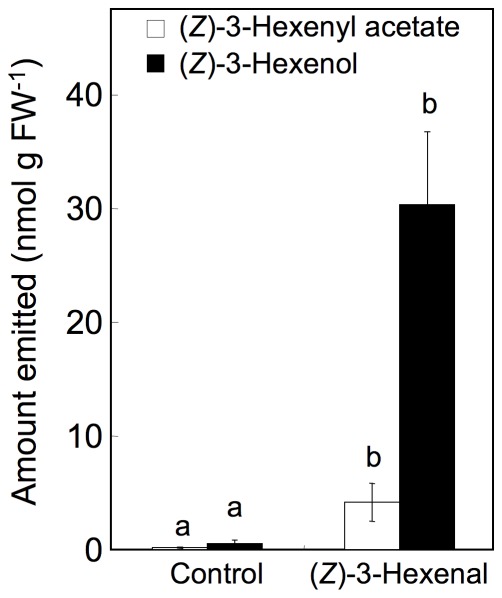
Conversion of (*Z*)-3-hexenal in intact *Arabidopsis* (*Col-0*). Intact *Arabidopsis* (*Col-0*) plants were exposed to (*Z*)-3-hexenal vapor (1.0 nmol cm^−3^), transferred into a fresh glass container, and the amounts of (*Z*)-3-hexenyl acetate (white bars) and (*Z*)-3-hexenol (black bars) emitted from the plants were quantified. Control plants were exposed to CH_2_Cl_2_ vapor (carrier solvent for volatiles). Error bars represent SE (*n* = 3). Data were analyzed by Tukey's test. Different letters above the bars indicate significant differences among treatments in the emission of each compound (*P*<0.05).

To estimate the ability of an intact *Arabidopsis* leaf to reduce (*Z*)-3-hexenal to (*Z*)-3-hexenol, we exposed intact leaves to (*Z*)-3-hexenal vapor. After exposure, the leaves were moved to a fresh container, and the amounts of GLVs emitted from the leaves were analyzed. In this experiment, we used *Arabidopsis* ecotype *Col-0*, because this ecotype has no HPL activity and few endogenous GLVs [22]. After the intact *Col-0* leaves were exposed to 1.0 nmol cm^−3^ (*Z*)-3-hexenal vapor for 30 min, we detected emissions of (*Z*)-3-hexenol and (*Z*)-3-hexenyl acetate, but not (*Z*)-3-hexenal (Fig. 3). This result suggested that most of the (*Z*)-3-hexenal that diffused into the intact leaves was reduced and/or acetylated sequentially to (*Z*)-3-hexenol and (*Z*)-3-hexenyl acetate. (*Z*)-3-Hexenal may have also been isomerized to (*E*)-2-hexenal and then subsequently reduced to yield *n*-hexanal by alkenal/one oxidoreductase [23], [24]; however, we did not detect saturated volatiles, and so this possibility was dismissed.

**Figure 4:**
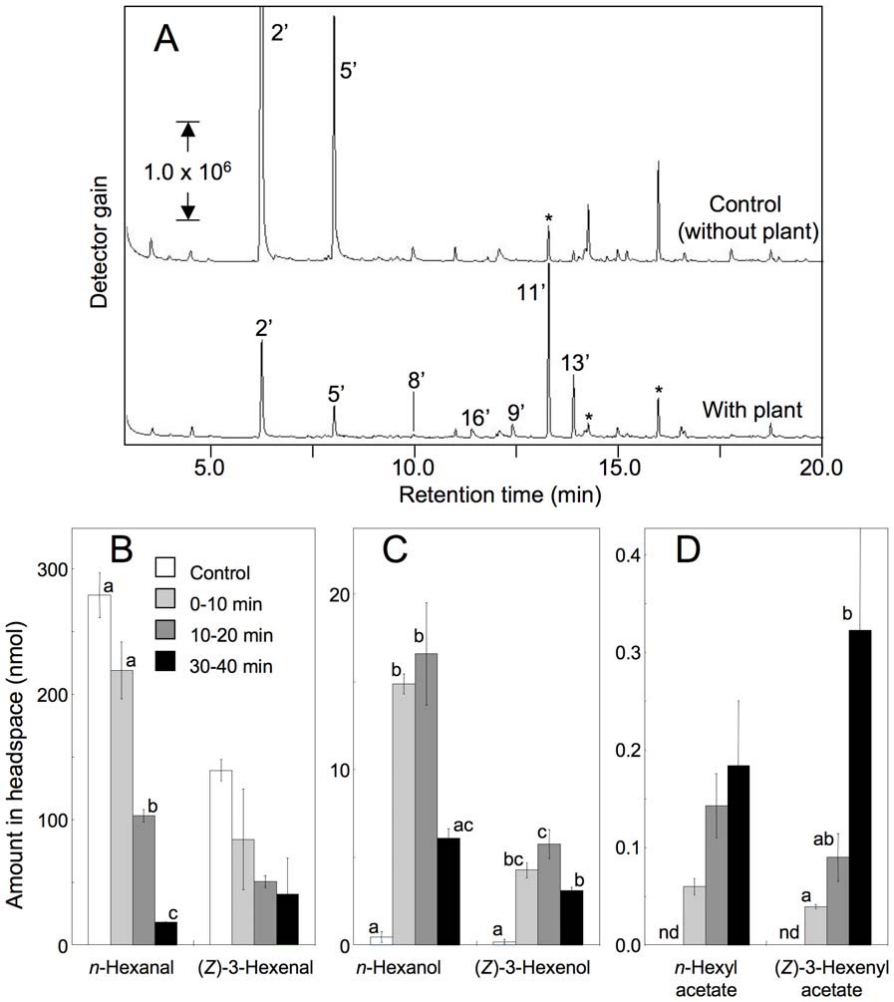
Conversion of U-^13^C-*n*-hexanal and U-^13^C-(*Z*)-3-hexenal over time by intact *Arabidopsis* plants. An intact *Arabidopsis* (*Col-0*) plant or a pot with soil (control) was placed in a plastic box with a cotton swab containing 2 µl of a mixture containing U-^13^C-*n*-hexanal and (*Z*)-3-hexenal. At 0, 10, or 30 min after the onset of exposure, an SPME fiber was positioned in the headspace of the box and volatiles were collected for 10 min. **A:** Typical chromatograms obtained after 40 min exposure. Compound numbers are as in the Fig. 2 legend. Numbers with prime marks indicate the corresponding ^13^C-labeled compound (>95% enrichment). The amounts of volatile aldehydes (**B**), volatile alcohols (**C**), and volatile acetates (**D**) were quantified from calibration curves constructed with authentic compounds. Error bars represent SE (*n* = 3). Data were analyzed by Tukey's test. Different letters above the bars indicate significant differences among the sampling times for each compound (*P*<0.05). nd: not detected.

Further evidence for the uptake and conversion of the volatile aldehydes and their subsequent re-emission by intact *Arabidopsis* leaves was obtained with U-^13^C-labeled *n*-hexanal and (*Z*)-3-hexenal with isotope enrichments of 98% (Fig. S3). An intact *Col-0* plant was exposed to vapor of ^13^C-labeled *n*-hexanal and (*Z*)-3-hexenal in a container, and the composition of volatiles in the headspace was analyzed after 10, 20, and 40 min (Fig. 4). The amount of *n*-hexanal decreased quickly within 40 min, and the amount of (*Z*)-3-hexenal showed the same tendency (Fig. 4B). The conversion of *n*-hexanal and (*Z*)-3-hexenal into *n*-hexanol and (*Z*)-3-hexenol was observed within 10 min after the onset of exposure. The amounts of alcohols remained almost constant over the next 10 min interval, and thereafter decreased over the next 20 min (Fig. 4C). In contrast, the amounts of *n*-hexyl acetate and (*Z*)-3-hexenyl acetate increased almost linearly until at least 40 min after the onset of exposure (Fig. 4D). The isotope enrichment in these derivatives, as calculated from the diagnostic m/z peaks of each compound, was 95% to 98% (Figs. S4, S5), which was almost the same as those of the *n*-hexanal and (*Z*)-3-hexenal (ca. 98%) used for the exposure. In the acetates, ^13^C was present only in the alkyl chain while the acetyl moiety contained ^12^C. This result indicated that the acetyl moiety was derived from endogenous acetyl-CoA. Thus, we concluded that almost all C6 volatiles emitted from exposed leaves originated from the labeled *n*-hexanal and (*Z*)-3-hexenal in the vapor phase of the container. Apparently, only a portion of *n*-hexanal and (*Z*)-3-hexenal was converted into their alcohols and esters within 20 min after exposure (Fig. 4B, 4C, and 4D). This result suggested that *n*-hexanal and (*Z*)-3-hexenal remained in the leaf tissues, or that they were converted into other non-volatile compounds, such as their glutathione conjugates [5], in the leaf tissues.

**Figure 5:**
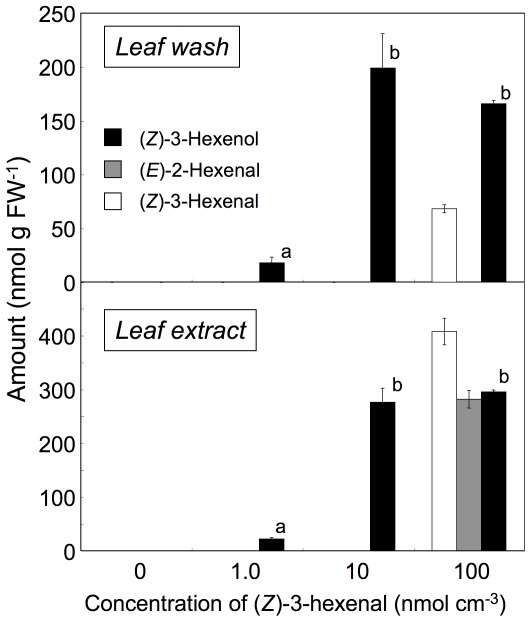
Localization of volatile compounds after exposure to (*Z*)-3-hexenal vapor. Intact leaves exposed to (*Z*)-3-hexenal vapor (0, 0.01, 0.1, and 1.0 mmol cm^−3^) were washed for 10 s with methanol to extract methanol-soluble surface volatiles (leaf wash) and the remaining surface-washed leaves were extracted with methyl *tert*-butyl ether (leaf extract). Error bars represent SE (*n* = 3). Data were analyzed by Tukey's test. Letters indicate significant differences in amounts of extracted compounds among exposure concentrations (*P*<0.05).

To investigate the site(s) of adsorption of the volatile compounds and to estimate the capacity of *Arabidopsis* leaves to reduce exogenously supplied (*Z*)-3-hexenal, the surfaces of intact *Col-0* leaves exposed to different concentrations of (*Z*)-3-hexenal for 10 min were washed with methanol (leaf wash) and then extracted with methyl *tert*-butyl ether (leaf extract) (Fig. 5). After exposing the leaves to 1.0 nmol cm^−3^ (*Z*)-3-hexenal, a significant amount of (*Z*)-3-hexenol was detected on leaf surfaces and in leaf tissues; however, (*Z*)-3-hexenal was not detected in either of these fractions. Acetates were not detected, probably because they were emitted quickly after formation. When ^13^C-labeled (*Z*)-3-hexenal and *n*-hexanal were used, the corresponding alcohols detected on the surface and in the tissues of leaves were exclusively derived from exogenously supplied C6-aldehydes (Fig. S6).

There was a greater amount of (*Z*)-3-hexenol detected in both leaf-surface and leaf-tissue fractions when 10 nmol cm^−3^ (*Z*)-3-hexenal was supplied, but (*Z*)-3-hexenal was still undetectable (Fig. 5). When 100 nmol cm^−3^ (*Z*)-3-hexenal was supplied, the amounts of (*Z*)-3-hexenol detected were almost equivalent to those found when 10 nmol cm^−3^ (*Z*)-3-hexenal was supplied, and substantial amounts of (*Z*)-3-hexenal were detected in both fractions. In the leaf extract, the isomerized form of (*Z*)-3-hexenal, i.e., (*E*)-2-hexenal, was also detected. This result suggested that (*Z*)-3-hexenal that diffused into the leaf tissues was immediately reduced into its corresponding alcohols, which then re-diffused out from the tissues through the surface into the atmosphere. The leaf tissues were able to reduce all of the exogenously supplied (*Z*)-3-hexenal within 10 min when it was supplied at 10 nmol cm^−3^, but not when it was supplied at higher concentrations. As a result, at higher concentrations of (*Z*)-3-hexenal exposure, a significant portion remained on the leaf surface or in the leaf tissues. In the leaf tissues, some of the remaining (*Z*)-3-hexenal was further isomerized to (*E*)-2-hexenal.

To examine the diffusion of volatile aldehydes from wounded tissues to neighboring intact tissues, we mechanically wounded the tips of *Col-0* leaves, and then placed an aqueous suspension of ^13^C-*n*-hexanal and (*Z*)-3-hexenal on the wounded sites. Five minutes after the treatment, the wounded half with the suspension was carefully excised with a razor blade, and the GLVs emitted from the remaining half were analyzed. This analysis allowed detection of ^13^C-*n*-hexanol and (*Z*)-3-hexenol and their acetates as well as ^13^C-*n*-hexanal and (*Z*)-3-hexenal (Table 1). C6-aldehydes were detected, probably because their local concentrations exceeded the rates of conversion. Again, the isotope enrichment of each molecule in the tissues was greater than 95%, indicating that all of the compounds detected in this experiment originated from the exogenously supplied compounds. These results indicated that some of the *n*-hexanal and (*Z*)-3-hexenal applied to the wounded region diffused out within 5 min to the neighboring intact leaf tissues, where they were converted to their alcohols and acetates.

**Table 1:** Diffusion of ^13^C-*n*-hexanal and (*Z*)-3-hexenal into neighboring intact tissues.

Compound^a^	Amount (nmol g fr wt^−1b^)
*n*-Hexanal	42.2±24.2
(*Z*)-3-Hexenal	13.0±10.6
*n*-Hexyl acetate	0.5±0.1
(*Z*)-3-Hexenyl acetate	0.7±0.2
*n*-Hexanol	57.5±23.2
(*Z*)-3-Hexenol	48.2±2.8

a Isotope enrichment of each compound was more than 95%.

b Values are mean ± SE (n = 3).

### Effect of co-factors on GLV composition

**Figure 6:**
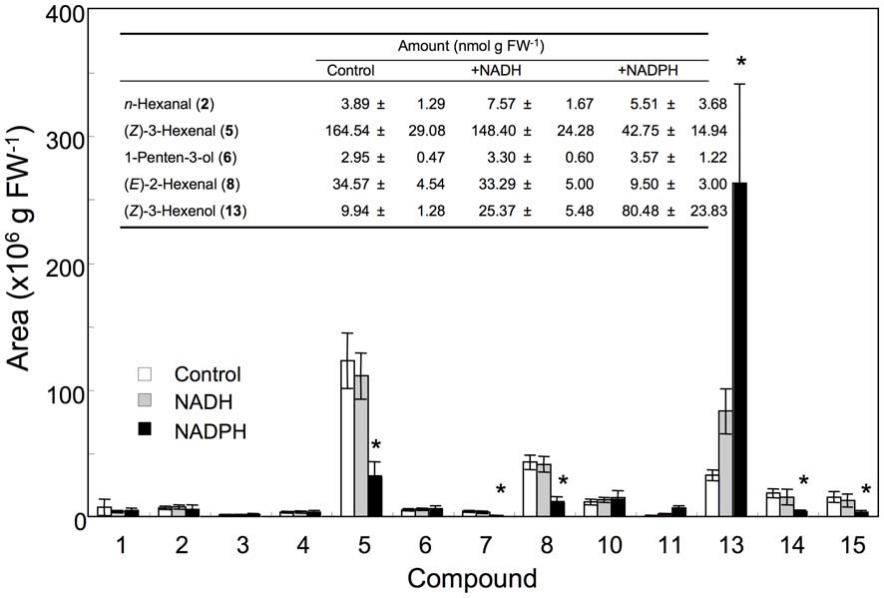
Conversion of volatile aldehydes to their corresponding alcohols in the presence of NADPH or NADH. *Arabidopsis* (*No-0*) leaves were disrupted completely in the absence (control, white bars) or presence of NADH (+NADH, shaded bars) or NADPH (+NADPH, black bars), and the volatile compounds that formed were analyzed. Inset: amounts of some volatile compounds quantified from corresponding calibration curves. Error bars represent SE (*n* = 3). Asterisks indicate significant differences compared with the control (*P*<0.05, *t*-test).

The preceding results suggested that there was a marked difference between disrupted and intact cells in their ability to reduce (*Z*)-3-hexenal. An electron donor is required for the enzymatic reduction of (*Z*)-3-hexenal to (*Z*)-3-hexenol. According to a previous report, the concentrations of NADH and NADPH in intact *Arabidopsis* leaves were 4–10 and 10 nmol g FW^−1^, respectively [25]. In our experiments, the concentration of (*Z*)-3-hexenol in partially disrupted leaves reached 145 nmol g FW^−1^ (Fig. 2); therefore, NADH and/or NADPH must have been regenerated to support the full reduction of the corresponding amounts of (*Z*)-3-hexenal in the cells. As a consequence, NADH or NADPH is likely to be the limiting factor in the ability of disrupted *Arabidopsis* leaf cells to reduce (*Z*)-3-hexenal. To confirm this possibility, we supplemented disrupted leaf cells with exogenous NADH or NADPH and examined the formation of volatiles by GC-MS analysis (Fig. 6). When NADPH was added, the amounts of (*Z*)-3-hexenal and (*E*)-2-hexenal decreased, whereas that of (*Z*)-3-hexenol increased. In contrast, NADH only slightly facilitated the conversion of (*Z*)-3-hexenal to the alcohol. This result confirmed that an NADPH-dependent enzyme was involved in the reduction of hexenals to their corresponding hexenols but all the hexenals formed in the disrupted tissues could not be reduced because of shortage of NADPH. A crude enzyme solution prepared from *Col-0* leaves also reduced (*Z*)-3-hexenal to (*Z*)-3-hexenol and showed 3.6-fold greater activity with NADPH than with NADH. The amount of *n*-hexanol was also slightly increased by the addition of NADPH, but the amounts of C5 alcohols such as 1-penten-3-ol and (*E*)-2-pentenol were barely changed. These results suggest that the reducing enzyme activated by the addition of NADPH was specific to C6 aldehydes. The specificity for C6 compounds observed here was unlike those of recombinant aldehyde reductases derived from *Arabidopsis*, which showed relatively higher activities with C5 compounds [24].

### Toxicity of (*Z*)-3-hexenal

**Figure 7:**
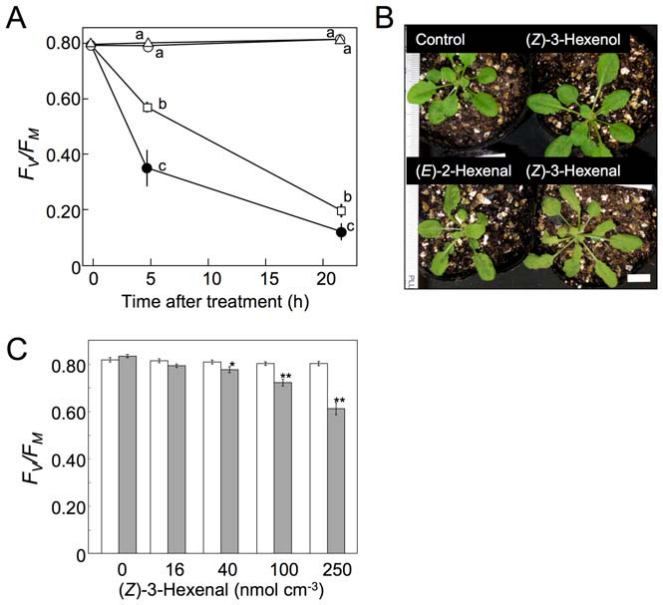
Changes in photosystem II activity in *Arabidopsis* leaves during exposure to C6-volatiles. **A:** *Arabidopsis* (*Col-0*) plants were exposed to vapor of CH_2_Cl_2_ (control, triangles), (*Z*)-3-hexenol (open circles), (*E*)-2-hexenal (squares), or (*Z*)-3-hexenal (closed circles) in the light (60 µmol m^−2^ s^−1^, supplied by white fluorescent lamps). PSII activity was determined using the chlorophyll fluorescence parameter *F_V_/F_M_*, detected with a MINI-PAM Chlorophyll Fluorometer. Plants were incubated in darkness for 5 min before each measurement. Error bars represent SE (*n* = 12). Data were analyzed by Tukey's test. Different letters indicate significant differences among treatments at different times (*P*<0.05). **B:** Representative plants after 22 h of exposure to the vapors. Note the yellowing and wilding of leaves exposed to (*E*)-2-hexenal and (*Z*)-3-hexenal. **C:** Plants were exposed to different concentrations of CH_2_Cl_2_ (control, withe bars) or (*Z*)-3-hexenal (shaded bars) for 10 min before *F_V_/F_M_* was measured. Significant differences between the control and the treated plants are indicated by asterisks (*, *P*<0.05; **, *P*<0.01; *t*-test).

One possible reason for the very rapid reduction of (*Z*)-3-hexenal to (*Z*)-3-hexenol using NADPH is that (*Z*)-3-hexenal is more toxic to plants than is (*Z*)-3-hexenol. To confirm this hypothesis, we exposed *Arabidopsis* plants to (*Z*)-3-hexenal, (*E*)-2-hexenal, and (*Z*)-3-hexenol vapors (100 nmol cm^−3^, equivalent to 2240 ppmV) and determined the chlorophyll fluorescence parameter *F_V_/F_M_* as an indicator of cell deterioration. When CH_2_Cl_2_ was used as a carrier solvent, the *F_V_/F_M_* value remained at its initial level throughout the experimental period (Fig. 7A). (*Z*)-3-Hexenol also had little effect. However, (*E*)-2-hexenal and (*Z*)-3-hexenal had marked effects, significantly decreasing the *F_V_/F_M_* value within 5 h after treatment. These negative effects were more pronounced 22 h after treatment. With (*E*)-2-hexenal and (*Z*)-3-hexenal exposure, patchy necrotic lesions appeared on the leaves by 5 h after treatment. By 22 h after treatment, parts of the leaves had yellowed and wilted (Fig. 7B). (*Z*)-3-Hexenal caused more visible symptoms than (*E*)-2-hexenal. It is possible that the *α*,*β*-unsaturated aldehydes, such as (*E*)-2-hexenal, HHE, and OHE/HPHE derived from (*Z*)-3-hexenal, were the ultimate causes of the toxic effects [26].

Plants were exposed to different concentration of (*Z*)-3-hexenal for 10 min, then *F_V_/F_M_* value was determined (Fig. 7C). At 16 nmol cm^−3^ of (*Z*)-3-hexenal, no significant decrease in PSII activity was observed, while at concentration at or above 40 nmol cm^−3^, significant decreases were observed. The range of (*Z*)-3-hexenal concentrations that was toxic to the plants (>40 nmol cm^−3^) roughly overlapped with the concentrations at which the plants failed to reduce all the aldehyde (ca. 100 nmol cm^−3^, cf. Fig. 5).

## Discussion

Exposing an intact *Arabidopsis* plant to a ^13^C-labeled volatile compound unambiguously indicated that the volatile aldehydes diffused into plant tissues from the atmosphere. Diffusion of (*Z*)-3-hexenal and *n*-hexanal from wounded sites into neighboring intact tissues was also evident in this study. The permeation and diffusion of (Z)-3-hexenal into plant tissues should be partly caused by the intrinsic chemical nature (i.e., moderate hydrophobicity with a logP value of 1.73) of the aldehydes [27]. Exogenously supplied (*Z*)-3-hexenal was quickly reduced in the intact tissues to yield (*Z*)-3-hexenol and (*Z*)-3-hexenyl acetate, as found in the partially wounded leaf tissues. This might explain why an increase in the amount of (*Z*)-3-hexenol contributed most to the variation in GLVs of *Arabidopsis* plants after infestation by chewing herbivores [28]. An intriguing question is why *Arabidopsis* harnesses such an energetically expensive reduction system in the intact region surrounding a wounded part. To address this question, one needs to understand the chemical nature of (*Z*)-3-hexenal and its related aldehydes.

(*Z*)-3-Hexenal itself has defensive functions against bacteria, fungi, and insects [6], [7], [8], [9]. Some (*Z*)-3-hexenal is further converted into (*E*)-2-hexenal spontaneously or enzymatically [1], [3], [4], and (*E*)-2-hexenal also has highly harmful effects on organisms because of its *α*,*β*-unsaturated carbonyl moiety [5], [29]. (*Z*)-3-Hexenal also has the potential to be oxygenated to yield OHE/HPHE or HHE (Fig. 2). These oxygenated unsaturated aldehydes are reactive species and have toxic effects on many organisms [30], [31]. Thus, the purpose of the rapid formation of (*Z*)-3-hexenal and related aldehydes at the wounded sites in plants is to defend the plants against pathogens and herbivores on an *ad hoc* basis. This conclusion is supported by the fact that the susceptibility of transgenic *Arabidopsis* plants with higher or lower HPL activities to a necrotrophic fungal pathogen, *Botrytis cinerea*, was largely correlated with the amounts of (*Z*)-3-hexenal and its related aldehydes they contained [8].

In the present study, we have shown that exposing *Arabidopsis* to (*Z*)-3-hexenal vapor damages leaf tissue, indicating that (*Z*)-3-hexenal (and its isomerized or oxygenated products) is also potentially harmful to the producer itself. The concentration of (*Z*)-3-hexenal used in this study (>40 nmol cm^−3^) was rather high; however, the local concentration of (*Z*)-3-hexenal at the disrupted tissues is expected to be higher (approx. 1.5 µmol cm^−3^ according to Fig. 2). Thus, we hypothesized that a plant must be able to cope with the (*Z*)-3-hexenal formed at the damaged site to avoid harming its own neighboring intact tissues. One way to detoxify (*Z*)-3-hexenal is to reduce it to (*Z*)-3-hexenol, which is less toxic. However, (*Z*)-3-hexenol is still an active compound in some instances, since it can induce a subset of defense genes [18]. Plants further convert (*Z*)-3-hexenol to (*Z*)-3-hexenyl acetate, which is even less active and more volatile (Henry's law constants for (*Z*)-3-hexenal, (*Z*)-3-hexenol, and (*Z*)-3-hexenyl acetate are approx. 6, 25, and 1 M atm^−1^, respectively). *Arabidopsis* can take advantage of the high volatility of (Z)-3-hexenyl acetate to eliminate it from plant tissues into the atmosphere [18]. Other detoxification systems are also possible. Glutathionylation could be involved, because high levels of GSH-adducts of reactive carbonyl species are found in plant tissues, especially those under stress [5], [32]. Some (*Z*)-3-hexenal and its derivatives could possibly also form adducts with proteins [30], [31]. Formation of these nonvolatile derivatives would account for the lower yields of C6 alcohols and acetates than expected from the decrease in the amount of C6 aldehydes during exposure (Fig. 4).

Our observations indicated that the conversion of (*Z*)-3-hexenal to (*Z*)-3-hexenol is catalyzed by an NADPH-dependent reductase that is abundant in the leaf tissues of *Arabidopsis*. The NADB_Rossman Superfamily in *Arabidopsis* consists of more than 50 genes. Some are involved in metabolism [33] and others are involved in detoxification of reactive chemicals [23], [24]. Recently, four members were shown to be involved in detoxification of reactive carbonyls in *Arabidopsis*. They showed relatively high activities with (*E*)-2-pentenal, while reduction of (*E*)-2-pentenal was barely observed in this study. The specific reductase involved in the formation of (*Z*)-3-hexenol from (*Z*)-3-hexenal still remains to be identified.

To support such efficient reduction, NADPH must be continuously regenerated from NADP^+^ via active primary metabolism in the intact cells neighboring the wounded, disrupted cells. Extensively disrupted *Arabidopsis* cells are less able to reduce (*Z*)-3-hexenal, because the regeneration of NADPH is no longer supported due to the lack of organized primary metabolism. When *Arabidopsis* plants were exposed to relatively low amounts (e.g., 10 nmol cm^−3^ used in this study) of (*Z*)-3-hexenal, they managed its toxicity by regenerating NADPH to reduce (*Z*)-3-hexenal to (*Z*)-3-hexenol. However, the plants suffered severe damage when the amounts of (*Z*)-3-hexenal exceeded their reduction capacity (e.g., 40–100 nmol cm^−3^), possibly because the regeneration of NADPH became insufficient. This idea was supported by the observation in this study that a significant portion of (*Z*)-3-hexenal remained, some of it isomerized to (*E*)-2-hexenal, in leaf tissues when the leaves were exposed to a high concentration (e.g., 100 nmol cm^−3^) of (*Z*)-3-hexenal.

Based on this study, we propose the following scenario for the rapid reduction of (*Z*)-3-hexenal: plants produce (*Z*)-3-hexenal at the damaged site for their direct defense. The surplus (*Z*)-3-hexenal produced at the damaged sites diffuses to the neighboring intact tissues, where it is then converted into (*Z*)-3-hexenol and (*Z*)-3-hexenyl acetate. The rapid conversion of (*Z*)-3-hexenal to (*Z*)-3-hexenol and (*Z*)-3-hexenyl acetate in intact tissues is important to minimize the toxic effects of (*Z*)-3-hexenal and its related aldehyde derivatives. To do this, plants employ energy-expensive biochemical reactions requiring NADPH and acetyl-CoA. Subsequently, the release of (*Z*)-3-hexenol and (*Z*)-3-hexenyl acetate from damaged leaves is used by herbivores, carnivores, and/or neighboring plants as infochemicals that indicate the presence of herbivores. Interestingly, accumulating evidence shows that (*Z*)-3-hexenol and (*Z*)-3-hexenyl acetate are more likely than (*Z*)-3-hexenal to function as infochemicals in indirect interactions in several plant species [34]. The ability to form GLVs has been conserved in the evolution and diversification of the plant kingdom. However, the intriguing question of why almost all plant species produce not only the aldehyde, but also the alcohol and acetate forms, of GLVs remains unanswered. The reason plants produce the alcohol and acetate forms as well as the aldehyde form of GLVs was partly explained by the toxicity of the aldehyde forms of GLVs. As a consequence, multiple forms of GLVs were available to be co-opted as information signals in plant-based interactions in ecosystems.

## Materials and Methods

### Plant Materials

*Arabidopsis thaliana* (L.) Heynh. (ecotype *No-0*, *Col-0*, *Ler*) plants were germinated in soil (Metro-Mix, Sun Gro Horticulture Distribution Inc., Bellevue, WA, USA) in plastic pots (6 cm i.d.). Plants were grown in a chamber at 22°C under fluorescent lights (60 µmol m^−2^ s^−1^) with a 14 h light/10 h dark photoperiod.

### Labeled compounds

The fatty acid mixture isolated from *Chlorella vulgaris* Beijerinck grown with ^13^CO_2_ was a generous gift from Chlorella Industry Co. (Tokyo, Japan). The fatty acid composition of the mixture was as follows: C16∶0 (25.2%), C16∶1 (1.7%), C16∶2 (6.5%), C16∶3 (4.6%), C18∶0 (1.2%), C18∶1 (13.4%), C18∶2 (29.7%), C18∶3 (10.0%), and unknown (7.7%). The isotope enrichment was 97.2%. C16∶2 and C18∶2 can be converted into *n*-hexanal, while C16∶3 and C18∶3 can be converted into (*Z*)-3-hexenal. The fatty acid mixture was sequentially reacted with soybean lipoxygenase-1 and recombinant bell pepper fruit hydroperoxide lyase [35]. After the reaction, CH_2_Cl_2_ extraction yielded a solution containing U-^13^C-*n*-hexanal and U-^13^C-(*Z*)-3-hexenal (Fig. S3). The isotope enrichments of *n*-hexanal (ratio between m/z 88 and 82) and (*Z*)-3-hexenal (ratio between m/z 104 and 98) were 99.6 and 98.8%, respectively. Even though the preparation contained other compounds, such as unreacted fatty acids, *n*-hexanal and (*Z*)-3-hexenal were the sole volatiles emitted from the preparation (see Fig. 5A).

### Volatile Analysis

The aboveground part of an *Arabidopsis* plant with fully developed leaves (37 days old) was cut off with a razor blade, weighed, and then carefully placed in a glass vial (22 ml, Perkin Elmer, Waltham, MA, USA) with 30 stainless beads (3 mm i.d.). When the volatiles in intact tissues were analyzed, 1 ml saturated CaCl_2_ solution was added to inactivate any enzymes present. The vial was sealed tightly with a butyl stopper and a crimp-top seal (National Scientific, Rockwood, TN, USA), and then the tissues were completely disrupted by vortexing the vial vigorously. To analyze the volatiles formed by completely disrupted leaves, the tissues were disrupted without addition of the saturated CaCl_2_ solution and incubated for 5 min at 25°C. After 5 min, 1 ml saturated CaCl_2_ solution was added to halt any enzyme reactions. When the effects of cofactors were examined, NADH or NADPH (Oriental Yeast Co., Tokyo, Japan; final concentration: 1 mM) was added to vials just before complete disruption with the beads. To evaluate volatiles formed in partially wounded leaves, wounds (approximately 20% of the leaf area) were made on every leaf with forceps without pinching the midvein. Immediately after wounding (within 10 sec), the aboveground part was cut and placed in the vial. After incubation at 25°C for 5 min, the tissues were disrupted with the stainless beads in the presence of 1 ml saturated CaCl_2_ solution.

An SPME fiber (50/30 µm DVB/Carboxen/PDMS, Supelco, Bellefonte, PA, USA) was exposed to the headspace of the vial for 30 min at 25°C. The fiber was inserted into the insertion port of a GC-MS (QP-5050, Shimadzu, Kyoto, Japan) equipped with a 0.25 µm×30 m Stabiliwax column (Restek, Bellefonte, PA, USA). The column temperature was programmed as follows: 40°C for 1 min, increasing by 15°C min^−1^ to 180°C for 1 min. The carrier gas (He) was delivered at a flow rate of 1 ml min^−1^. The glass insert was an SPME Sleeve (Supelco). Splitless injection with a sampling time of 1 min was used. The fiber was held in the injection port for 10 min to fully remove any compounds from the matrix. The temperatures of the injector and interface were 200 and 230°C, respectively. The mass detector was operated in the electron impact mode with ionization energy of 70 eV. To identify each compound, we used retention indices and MS profiles of corresponding authentic specimens. For quantification, aqueous solutions of each compound (suspended in the presence of a small amount of Tween 20) were mixed together at different ratios and then mixed with 1 ml saturated CaCl_2_ solution in a glass vial. The volatiles were analyzed with SPME-GC-MS as described above, and a calibration curve for each compound was constructed. We used the same fiber during analyses. (*Z*)-3-hexenal was a generous gift from Zeon Co. (Tokyo, Japan). HHE was purchased from Cayman Chemical Co. (Ann Arbor, MI, USA). Other volatiles were purchased from Wako Pure Chemicals (Osaka, Japan). Measurements were carried out on at least three replicates with different plants.

The amounts of fatty acids in *Arabidopsis* leaves were determined after extraction of the fully developed *Col-0* leaves with CHCl_3_/methanol (2∶1, v/v) and subsequent transmethylation with 5% HCl-methanol. The fatty acids quantities were determined with GC analysis using margaric acid as an internal standard. To determine the (*Z*)-3-hexenal-reductase activity *in vitro*, *Arabidopsis Col-0* leaves were homogenized with 2 vol (v/w) 10 mM Tris-Cl, pH 7.5, containing 1 mM phenylmethansulfonylfluoride, then, centrifuged at 20,000× g for 10 min at 4°C. The supernatant was used as the crude enzyme solution. The reaction was carried out with 4 mM (*Z*)-3-hexenal in the presence of 5 mM NADPH or NADH for 1 h at 37°C. The volatile products were analyzed with a headspace-GC system as described previously [36].

### Volatile Treatment of Plants

*Arabidopsis Col-0* plants (41 days old) grown in pots were placed in plastic boxes (340 cm^3^) with cotton swabs. A small amount (3.4 µl) of 0.1 M (*Z*)-3-hexenal dissolved in CH_2_Cl_2_ was applied to the cotton swab and the box was tightly sealed. After exposure to the vapor for 30 min at 25°C in the light (60 µmol m^−2^ s^−1^), the plant was removed from the box. Then, the aboveground part was cut off with a razor blade and placed in a glass vial (61 cm^3^). The cut surface of the stem was immersed in water (2 ml) at the bottom of the vial. The vial was tightly closed and SPME-GC-MS analysis was performed as described above without crushing the leaf tissues. Subsets of the plants were treated with abscisic acid by drenching the soil with 50 ml of 100 µM abscisic acid until the solution ran from the bottom of the pot [25]. The plant was used for vapor exposure analysis after 40 min.

To examine the time-course of adsorption and conversion of *n*-hexanal and (*Z*)-3-hexenal, an *Arabidopsis Col-0* plant was placed in a plastic box (340 cm^3^) with a cotton swab to which 2 µl of a mixture containing 1.1 mM U-^13^C-*n*-hexanal and 2.4 mM (*Z*)-3-hexenal had been applied. The SPME fiber was inserted either immediately or at 10 or 30 min after exposure, and the volatiles in the headspace were collected for an additional 10 min. To examine the diffusion of the volatile aldehydes into leaf tissues, an intact *Col-0* plant was exposed to (*Z*)-3-hexenal at 1.0, 10, and 100 nmol cm^−3^ (or a mixture consisting of U-^13^C-*n*-hexanal and (*Z*)-3-hexenal at 0.31 and 0.69 nmol cm^−3^, respectively), as described above, for 10 min at 25°C. Then, the leaves were cut at the base of the petiole, weighed, and washed for 10 s in 4 ml methanol containing 22.1 µg nonyl acetate (IS). After towel-blotting, the remaining leaf material was extracted with 2 ml methyl *tert*-butyl ether containing 22.1 µg nonyl acetate by heating at 60°C for 10 min. The leaf-wash and leaf-extract were directly analyzed by GC-MS as described previously. To examine diffusion of *n*-hexanal and (*Z*)-3-hexenal from wounded parts into neighboring intact tissues, two wounds (2 mm wide×7 mm long, alongside the costa) were made at the tips of each of six fully developed *Col-0* leaves with forceps, and droplets (2 µl) of the aqueous mixture containing 1.5 mM U-^13^C-*n*-hexanal and 3.3 mM (*Z*)-3-hexenal were placed at the basal edge of the wounds. After incubation for 5 min at 25°C, the leaf tips with the droplets were cut off with a razor blade, and then the volatiles emitted from the remaining parts of leaves were collected with an SPME fiber in a closed glass vial (61 cm^3^) for 30 min at 25°C.

The maximum quantum yield of photosystem II (PSII) was estimated from chlorophyll fluorescence measurements using a pulse amplitude modulated (PAM) fluorometer (Mini-PAM photosynthesis yield analyzer; Walz, Effeltrich, Germany). The yield of PSII was calculated as the ratio of *F*_V_/*F*_M_ [37]. The saturation pulse duration was 0.8 s with an intensity level of approximately 8300 µmol m^−2^ s^−1^. Three pots of *Col-0* plants (60 days old) were placed inside a glass cylinder (1,000 cm^3^), and 100 µl CH_2_Cl_2_ or a 1 M CH_2_Cl_2_ solution of (*Z*)-3-hexenal, (*E*)-2-hexenal, or (*Z*)-3-hexenol was applied to a cotton swab placed 5 cm above the canopy of the plants. The flask was sealed with a polyvinylidenechloride sheet and incubated at 22°C in the light (60 µmol m^−2^ s^−1^). For each plant, the fluorescence measurements were conducted at the midpoint of four fully-mature leaves. The plants were dark-acclimated for at least 5 min before PAM analysis. In order to examine the dose response, the plants were exposed to 16, 40, 100, or 250 nmol cm^−3^ of (*Z*)-3-hexenal for 10 min in a glass cylinder, then the *F*_V_/*F*_M_ values were determined.

## Supporting Information

Figure S1 **Total ion chromatogram of SPME-adsorbed volatiles collected from intact, partially wounded, and completely disrupted leaves.** Peaks: 1, 1-penten-3-one; 2, *n*-hexanal; 3, 2-pentenal*; 4, 2-pentenal* (probably a geometrical isomer of compound 3); 5, (*Z*)-3-hexenal; 6, 1-penten-3-ol; 7, (*Z*)-2-hexenal*; 8, (*E*)-2-hexenal; 9, (*Z*)-3-hexenyl acetate; 10, (*Z*)-2-pentenol; 11, *n*-hexanol; 12, (*E*)-3-hexenol*; 13, (*Z*)-3-hexenol; 14, 4-oxo-(*E*)-2-hexenal*; 15, 4-hydroxy-(*E*)-2-hexenal. Asterisks indicate compounds that were tentatively identified based on their MS data (according to the NIST library). Compounds without asterisks were identified based on retention indices and MS of corresponding authentic specimens.
(TIF)

Figure S2 **MS profiles of authentic HHE and two volatiles from disrupted leaves.** **A:** Authentic 4-hydroxy-(*E*)-2-hexenal (HHE); **B:** Peak 15 (Rt at 19.88 min) from Figure S1 was identified as HHE; **C:** Peak 14 (Rt at 15.48 min) in Figure S1 was tentatively identified as 4-oxo-(*E*)-2-hexenal (OHE) based on the MS profile of OHE reported elsewhere [38].
(TIF)

Figure S3 **MS profiles of U-^13^C-labeled** ***n*** **-hexanal and (** ***Z*** **)-3-hexenal.** The MS profiles of ^12^C-*n*-hexanal and (*Z*)-3-hexenal in the NIST database are shown for comparison. Red arrows indicate ion peaks used to calculate isotope enrichment.
(TIF)

Figure S4 **MS profiles of U-^13^C-labeled (** ***Z*** **)-3-hexenol and (** ***Z*** **)-3-hexenyl acetate.** The MS profiles of ^12^C-(*Z*)-3-hexenol and (*Z*)-3-hexenylacetate in the NIST database are shown for comparison. Red arrows indicate ion peaks used to calculate isotope enrichment.
(TIF)

Figure S5 **MS profiles of U-^13^C-labeled** ***n*** **-hexanol and** ***n*** **-hexyl acetate.** The MS profiles of ^12^C-*n*-hexanol and *n*-hexyl acetate in the NIST database are shown for comparison. Red arrows indicate ion peaks used for to calculate isotope enrichment.
(TIF)

Figure S6 **Localization of volatile compounds after exposure.** Intact leaves exposed to vapor of U-^13^C-*n*-hexanal and (*Z*)-3-hexenal were washed for 10 s with methanol to extract methanol-soluble surface volatiles and the remaining surface-washed leaves were extracted with methyl *tert*-butyl ether, and then amounts of volatile compounds originating from U-^13^C-n-al and Z3al were quantified.
(TIF)
