# Supplementary figures and images for: Differential Metabolisms of Green Leaf Volatiles in Injured and Intact Parts of a Wounded Leaf Meet Distinct Ecophysiological Requirements
Source: PLoS One. 2012 Apr 30;7(4):e36433. doi: 10.1371/journal.pone.0036433 (PMC3340338; doi:10.1371/journal.pone.0036433)

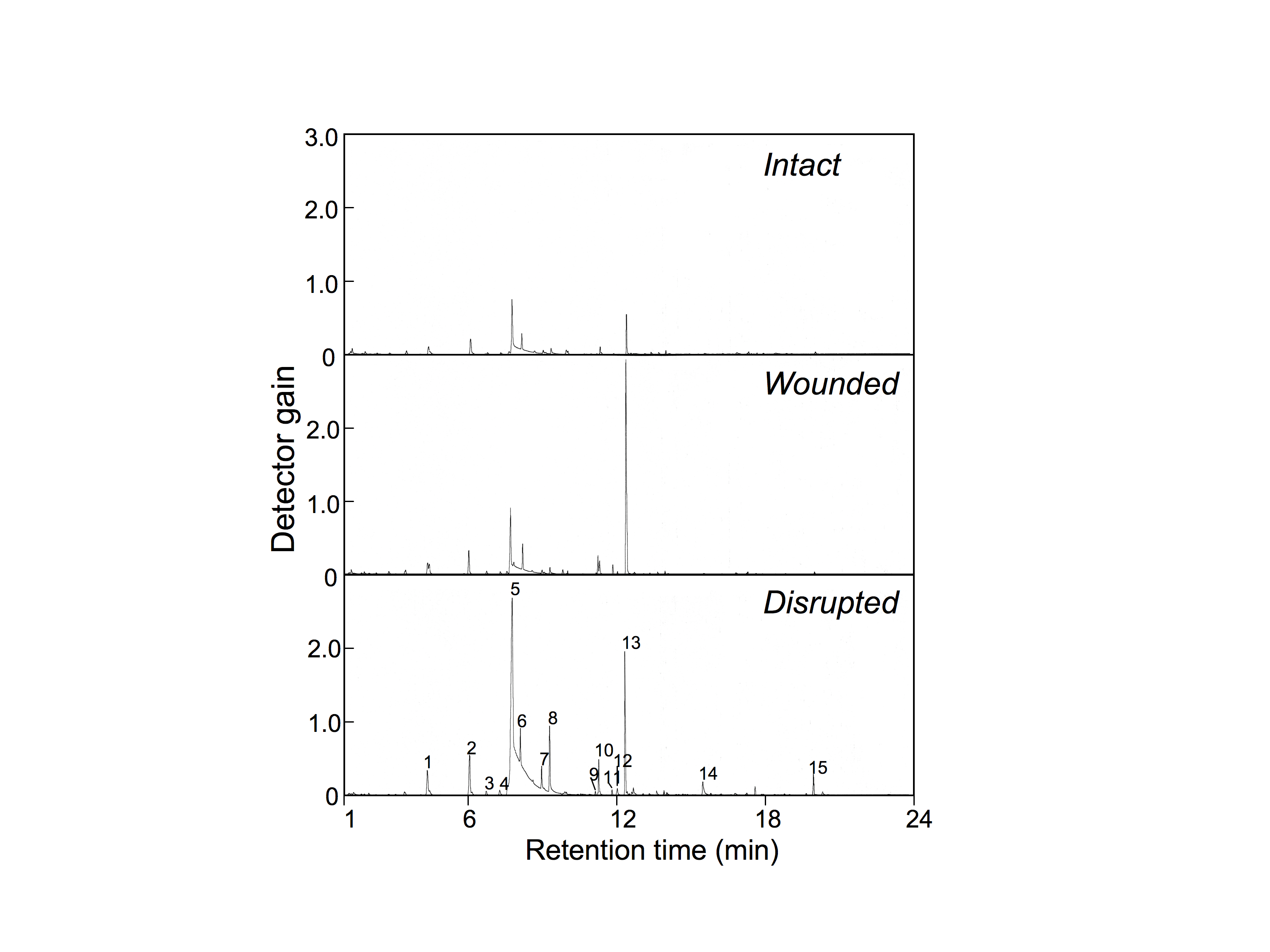

Supplement: Figure S1 — Total ion chromatogram of SPME-adsorbed volatiles collected from intact, partially wounded, and completely disrupted leaves. Peaks: 1, 1-penten-3-one; 2, n-hexanal; 3, 2-pentenal*; 4, 2-pentenal* (probably a geometrical isomer of compound 3); 5, (Z)-3-hexenal; 6, 1-penten-3-ol; 7, (Z)-2-hexenal*; 8, (E)-2-hexenal; 9, (Z)-3-hexenyl acetate; 10, (Z)-2-pentenol; 11, n-hexanol; 12, (E)-3-hexenol*; 13, (Z)-3-hexenol; 14, 4-oxo-(E)-2-hexenal*; 15, 4-hydroxy-(E)-2-hexenal. Asterisks indicate compounds that were tentatively identified based on their MS data (according to the NIST library). Compounds without asterisks were identified based on retention indices and MS of corresponding authentic specimens. (TIF) [file pone.0036433.s001.tif]

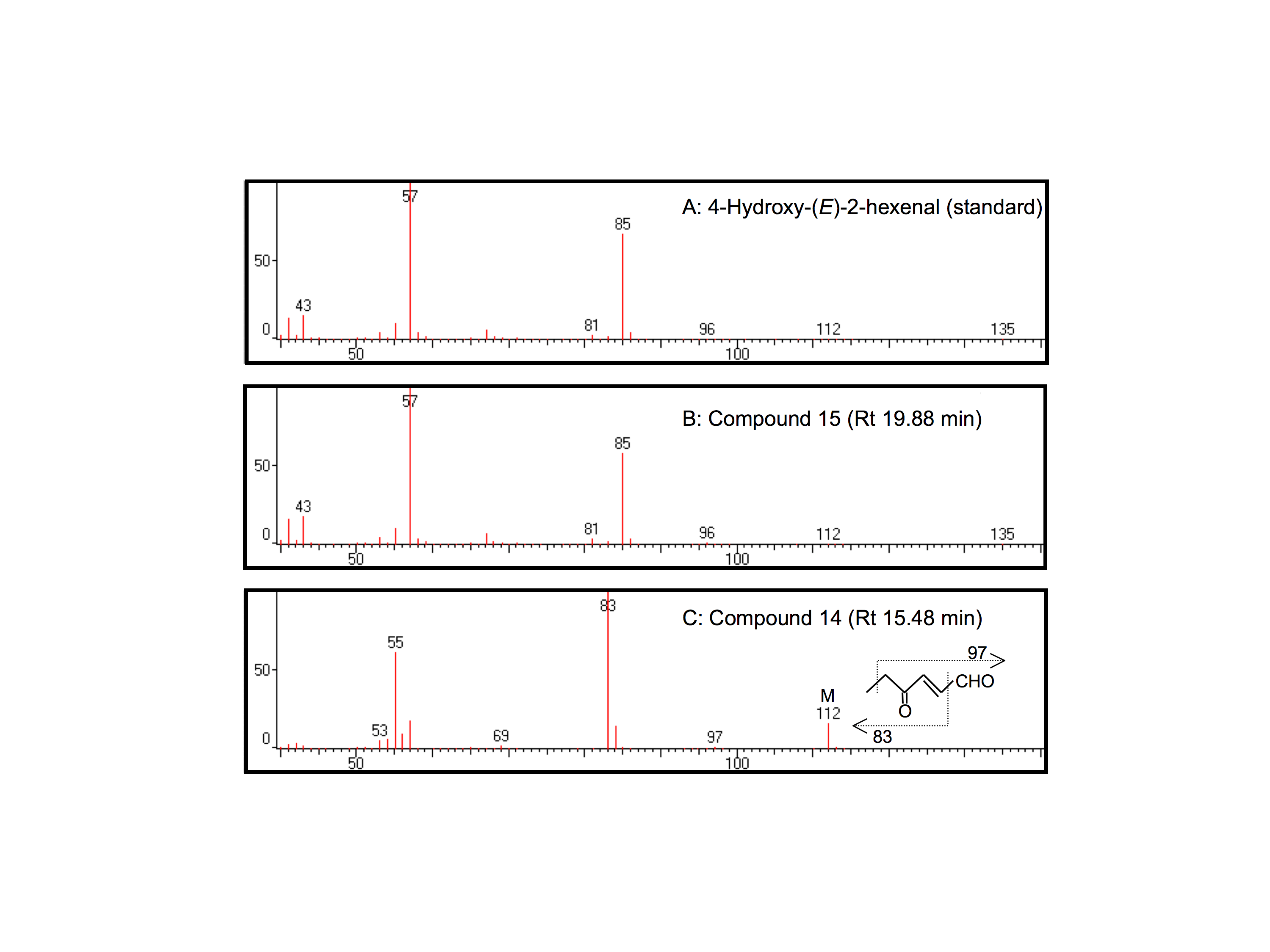

Supplement: Figure S2 — MS profiles of authentic HHE and two volatiles from disrupted leaves. A: Authentic 4-hydroxy-(E)-2-hexenal (HHE); B: Peak 15 (Rt at 19.88 min) from Figure S1 was identified as HHE; C: Peak 14 (Rt at 15.48 min) in Figure S1 was tentatively identified as 4-oxo-(E)-2-hexenal (OHE) based on the MS profile of OHE reported elsewhere [38]. (TIF) [file pone.0036433.s002.tif]

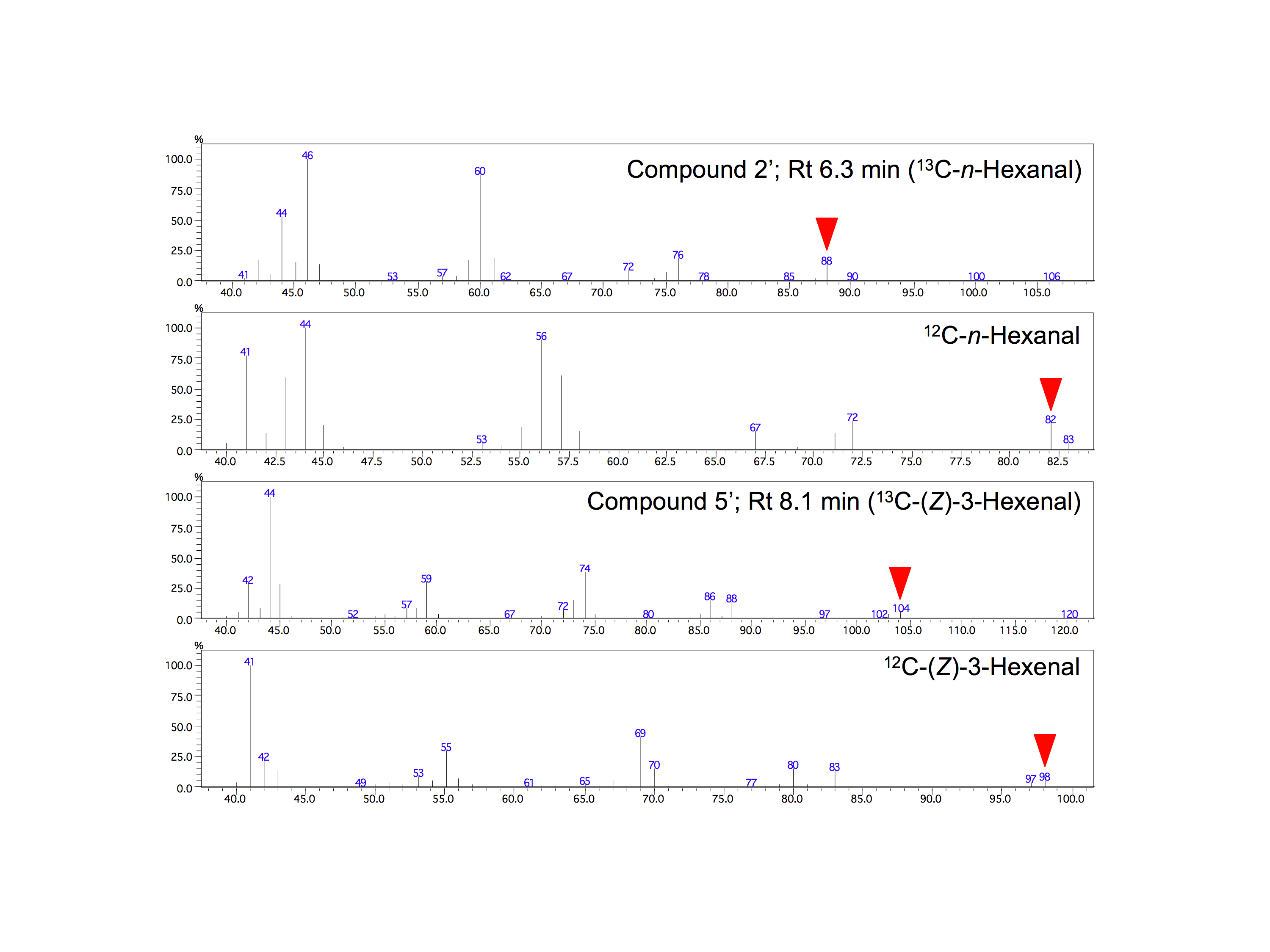

Supplement: Figure S3 — MS profiles of U-13C-labeled n -hexanal and ( Z )-3-hexenal. The MS profiles of 12C-n-hexanal and (Z)-3-hexenal in the NIST database are shown for comparison. Red arrows indicate ion peaks used to calculate isotope enrichment. (TIF) [file pone.0036433.s003.tif]

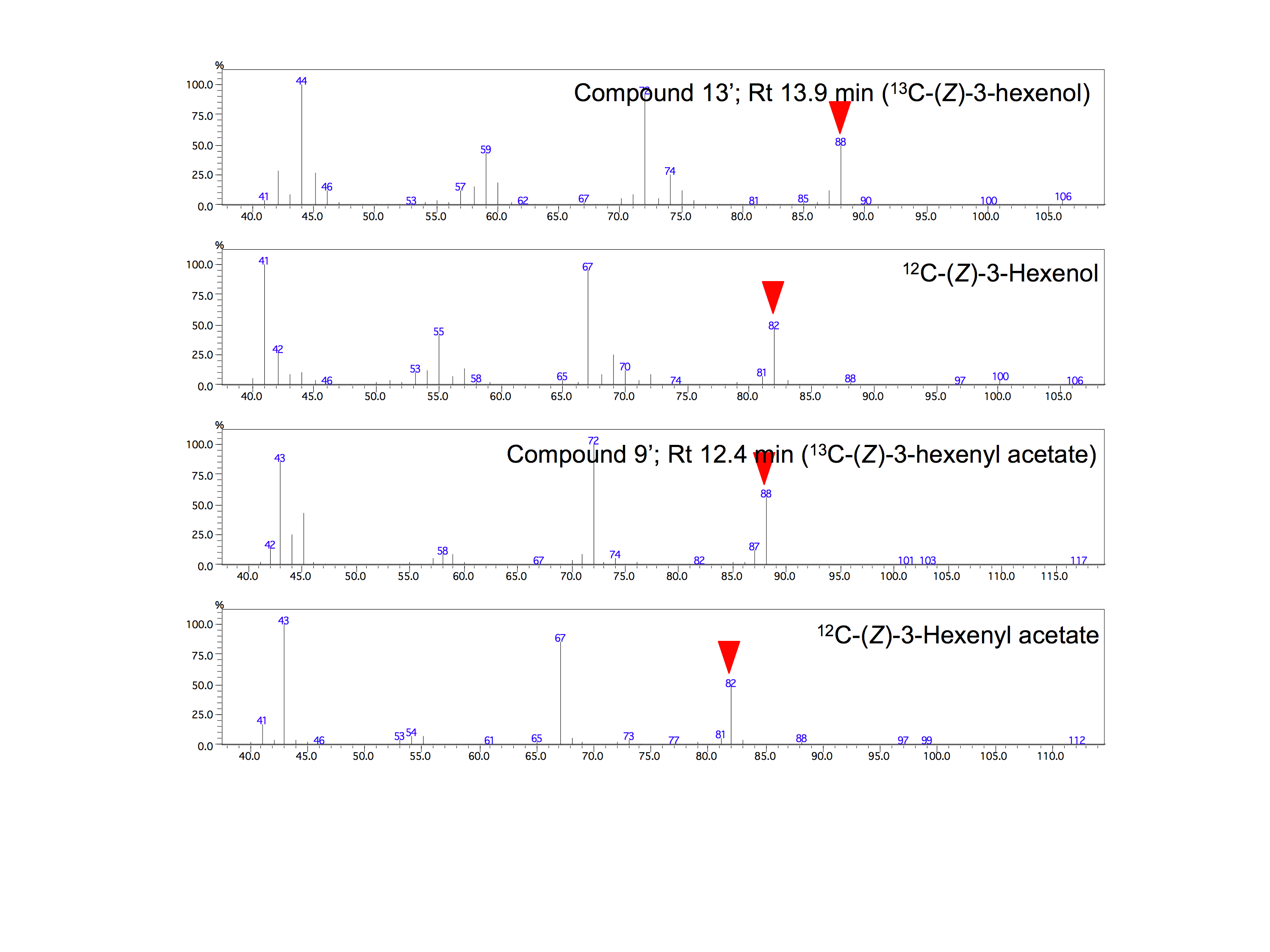

Supplement: Figure S4 — MS profiles of U-13C-labeled ( Z )-3-hexenol and ( Z )-3-hexenyl acetate. The MS profiles of 12C-(Z)-3-hexenol and (Z)-3-hexenylacetate in the NIST database are shown for comparison. Red arrows indicate ion peaks used to calculate isotope enrichment. (TIF) [file pone.0036433.s004.tif]

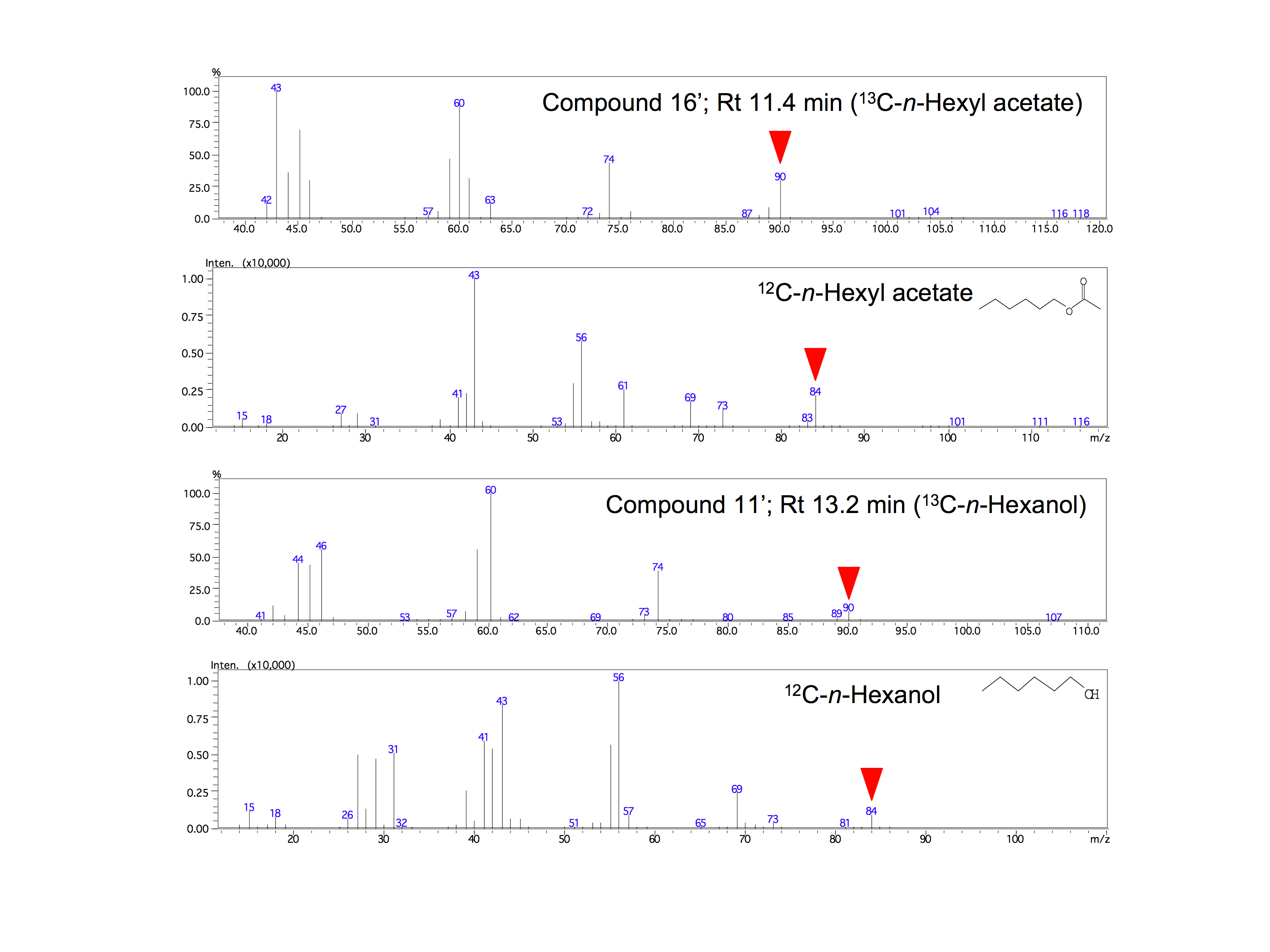

Supplement: Figure S5 — MS profiles of U-13C-labeled n -hexanol and n -hexyl acetate. The MS profiles of 12C-n-hexanol and n-hexyl acetate in the NIST database are shown for comparison. Red arrows indicate ion peaks used for to calculate isotope enrichment. (TIF) [file pone.0036433.s005.tif]

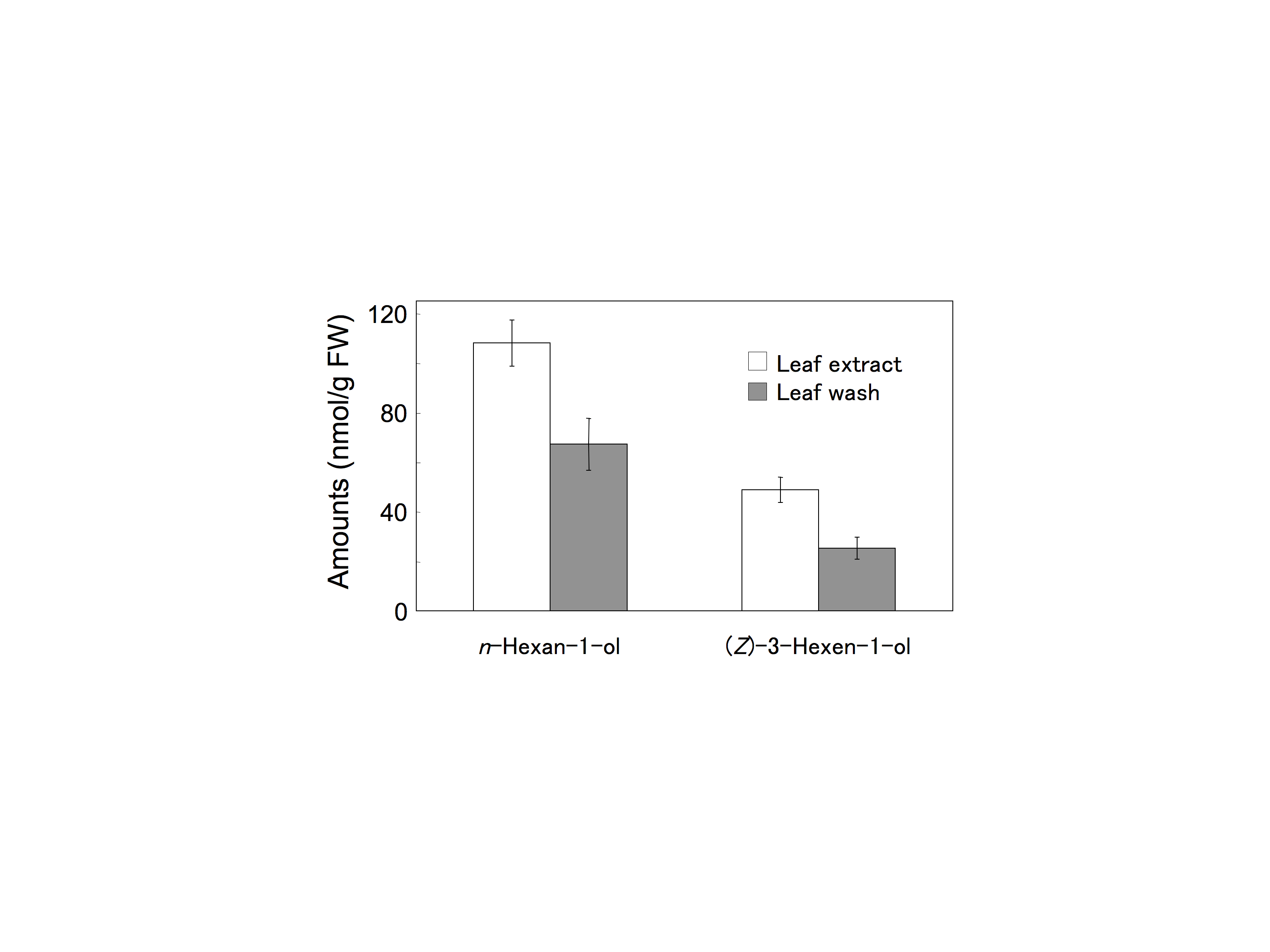

Supplement: Figure S6 — Localization of volatile compounds after exposure. Intact leaves exposed to vapor of U-13C-n-hexanal and (Z)-3-hexenal were washed for 10 s with methanol to extract methanol-soluble surface volatiles and the remaining surface-washed leaves were extracted with methyl tert-butyl ether, and then amounts of volatile compounds originating from U-13C-n-al and Z3al were quantified. (TIF) [file pone.0036433.s006.tif]
